# EspJ of enteropathogenic and enterohaemorrhagic *Escherichia coli* inhibits opsono-phagocytosis

**DOI:** 10.1111/j.1462-5822.2007.01112.x

**Published:** 2008-05-01

**Authors:** Oliver Marchès, Valentina Covarelli, Sivan Dahan, Céline Cougoule, Pallavi Bhatta, Gad Frankel, Emmanuelle Caron

**Affiliations:** 1 Division of Cell and Molecular Biology, Flowers Building, Imperial College London London SW7 2AZ, UK; 2 Centre for Molecular Microbiology and Infection, Flowers Building, Imperial College London London SW7 2AZ, UK

## Abstract

A key strategy in microbial pathogenesis is the subversion of the first line of cellular immune defences presented by professional phagocytes. Enteropathogenic and enterohaemorrhagic *Escherichia coli* (EPEC and EHEC respectively) remain extracellular while colonizing the gut mucosa by attaching and effacing mechanism. EPEC use the type three secretion system effector protein EspF to prevent their own uptake into macrophages. EPEC can also block *in trans* the internalization of IgG-opsonized particles. In this study, we show that EspJ is the type three secretion system effector protein responsible for *trans*-inhibition of macrophage opsono-phagocytosis by both EPEC and EHEC. While EspF plays no role in *trans*-inhibition of opsono-phagocytosis, *espJ* mutants of EPEC or EHEC are unable to block uptake of opsonized sheep red blood cells (RBC), a phenotype that is rescued upon complementation with the *espJ* gene. Importantly, ectopic expression of EspJ^EHEC^ in phagocytes is sufficient to inhibit internalization of both IgG- and C3bi-opsonized RBC. These results suggest that EspJ targets a basic mechanism common to these two unrelated phagocytic receptors. Moreover, EspF and EspJ target independent aspects of the phagocytic function of mammalian macrophages *in vitro*.

## Introduction

Enteropathogenic and enterohaemorrhagic *Escherichia coli* (EPEC and EHEC respectively) belong to a family of medically important diarrhoeagenic pathogens, which colonize the gut mucosa by the attaching and effacing (A/E) mechanism (for review, see Kaper *et al*., 2004). The genes responsible for the A/E phenotype are carried on the locus of enterocyte effacement (LEE) pathogenicity island (McDaniel *et al*., 1995), which encodes transcriptional regulators, the adhesin intimin (Jerse *et al.*, 1990), structural components of a type three secretion system (T3SS) (Jarvis *et al*., 1995), chaperones, as well as translocator and effector proteins (reviewed in Garmendia *et al*., 2005). A/E lesions are characterized by localized destruction of the brush border microvilli and intimate attachment of the bacteria to the apical membrane of enterocytes (Knutton *et al*., 1987).

EPEC and EHEC use the T3SS to inject into mammalian host cells dozens of effector proteins (Garmendia *et al*., 2005; Spears *et al*., 2006; Tobe *et al*., 2006), which target different subcellular compartments and affect diverse signalling pathways and physiological processes. Among the effector proteins are EspI/NleA which is targeted to the Golgi apparatus (Gruenheid *et al*., 2004; Mundy *et al*., 2004), EspG and EspG2 which disrupt the microtubule network (Matsuzawa *et al*., 2004; Hardwidge *et al*., 2005; Shaw *et al*., 2005a; Tomson *et al*., 2005); EspF which is targeted to the mitochondria and involved in disruption of the tight junction barrier, elongation of the intestinal brush border microvilli and cell death (Crane *et al*., 2001; McNamara *et al*., 2001; Nougayrède and Donnenberg, 2004; Nagai *et al*., 2005; Shaw *et al*., 2005b); Map which induces filopodia formation (Kenny *et al*., 2002); and Tir which downregulates Map-induced signals (Kenny *et al*., 2002) and is involved in extensive remodelling of the intermediate filament and the actin microfilament networks (Kenny *et al*., 1997; reviewed in Caron *et al*., 2006).

Avoidance of phagocytosis and the undermining of macrophage signalling are common strategies used by pathogenic bacteria to colonize the host while evading immune defences (Coombes *et al*., 2004). Phagocytosis is the process by which macrophage, neutrophils and dendritic cells internalize particulate material over 0.5 μm in diameter. Phagocytic uptake is a multistep, zipper-like process, initiated by the ligation of surface receptors and driven by a local remodelling of the actin cytoskeleton. The two best-characterized phagocytic receptors, complement receptor 3 (CR3) and Fc gamma receptors (FcγR), bind to opsonins deposited onto their targets, respectively, complement fragment C3bi and IgG. These two receptors are thought to mediate most of the phagocytic events occurring during innate and adaptive immune responses. Despite a conservation in principles, the mechanisms of internalization through CR3 and FcγR are known to involve different mechanisms and signalling pathways (Allen and Aderem, 1996; Caron and Hall, 1998).

EPEC and EHEC colonize the gut epithelium while remaining extracellular. Interestingly, EPEC is able to block its own uptake by professional phagocytes, a process we refer to as *cis*-inhibition of phagocytosis (Goosney *et al*., 1999; Celli *et al*., 2001; Quitard *et al*., 2006). The mechanism involved depends on the T3SS effector EspF but is poorly understood, although subversion of a phosphatidyl inositol 3-kinase (PI3K)-controlled pathway has been invoked (Celli *et al*., 2001; Quitard *et al*., 2006). Importantly, EPEC were also reported to inhibit *in trans* the phagocytosis of IgG-opsonized zymosan particles via the FcγR upon infection of macrophages (Celli *et al*., 2001), although the mechanism involved is not known. The aim of this study was to investigate the basis for EPEC O127:H6 *trans*-antiphagocytic activity and to determine if the same mechanism is shared with EHEC O157:H7, which is the most common virulent EHEC serotype.

## Results

### EPEC inhibit opsono-phagocytosis in a T3SS-dependent mechanism

**Fig. 1:**
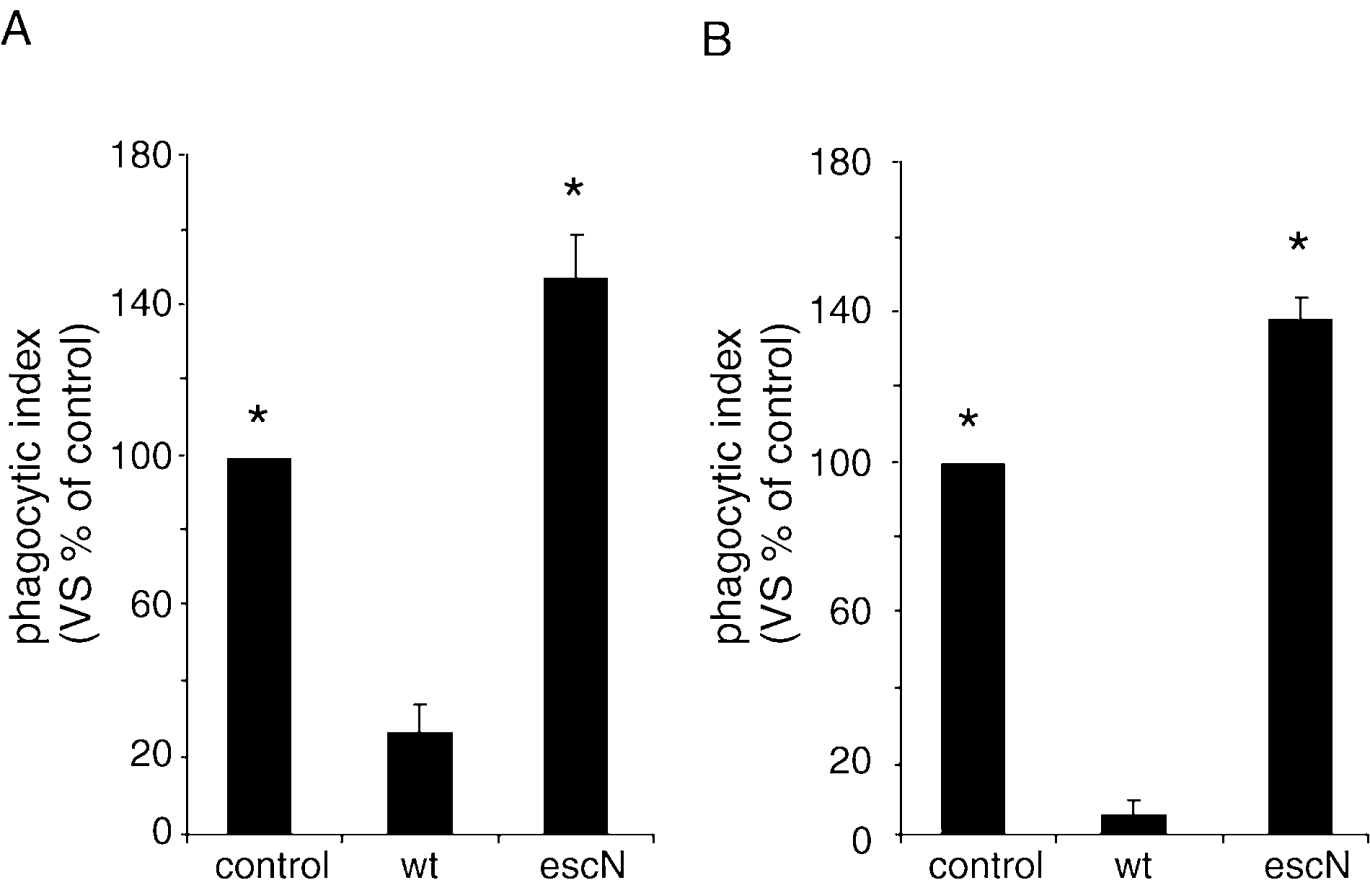
EPEC inhibit FcγR-mediated phagocytosis in a T3SS-dependent manner. J774.A1 macrophages (A) or FcγR-transfected Cos cells (B) were left uninfected (control) or infected with primed wild-type (wt) and Δ*escN* (escN) EPEC strains for 1 h and then challenged with IgG-RBC for 30 min. Cells were processed for immunofluorescence and scored for phagocytic index, i.e. the number of RBC bound to 100 cells. Values are expressed relative to the non-infected control (none) values which were set up at 100. Results are the mean ± SD of two independent experiments. Asterisks (*) denote a statistically significant difference with the wild-type strain.

J774.A1 macrophages were infected for 1 h with wild-type EPEC O127:H6 (strain E2348/69) and in parallel with its isogenic T3SS-deficient EPECΔ*escN* mutant (strain ICC192) (Garmendia *et al*., 2004). Uninfected macrophages were used as control. To maximize LEE gene expression EPEC strains were primed for 3 h in Dulbecco's modified Eagle's medium (DMEM) prior to infection (Collington *et al*., 1998). In order to assist detection of bacteria in infected macrophages, strains were transformed with a GFP-expressing plasmid (pFVp25.1). After infection, macrophages were washed then challenged with sheep red blood cells (RBC) pre-opsonized with either rabbit IgG or IgM and C5-deficient serum, to direct RBC for phagocytosis through FcγR and CR3 respectively (Caron and Hall, 1998). Macrophages were then fixed and RBC differentially labelled pre- and post-permeabilization in order to discriminate extracellular from phagocytosed RBC. Infection of J774.A1 macrophages with wild-type EPEC dramatically reduced uptake of both IgG- and C3bi-opsonized RBC (Fig. 1). In contrast, infection with the *escN* mutant resulted in phagocytosis of RBC at a comparable level to the non-infected control (Figs 1A and 3B).

Cos-7 cells transfected with phagocytic receptors provide an alternative model to study opsono-phagocytosis in isolation from macrophage receptors and secreted products (Caron and Hall, 1998). In order to confirm the results observed in J774.A1, Cos-7 cells were transfected with a construct encoding human FcγRIIA, which confers strong IgG-dependent phagocytic ability; then infected with EPEC strains and challenged with IgG-opsonized RBC. Infection with wild-type EPEC abrogated phagocytosis of opsonized RBC, as seen in J774.A1 macrophages. In contrast, when infected with EPECΔ*escN*, efficient phagocytosis of RBC via the FcγR was observed (Fig. 1B).

### EspJ mediates EPEC *trans*-antiphagocytic activity

**Fig. 2:**
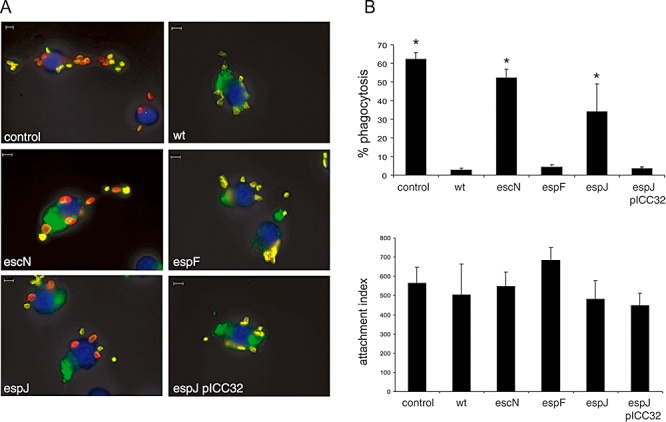
EPEC inhibition of FcγR-mediated phagocytosis is EspF-independent but requires translocation of EspJ. J774.A1 macrophages were left uninfected (control) or infected for 1 h with primed wild-type (wt), Δ*escN* (escN), Δ*espF* (espF), Δ*espJ* (espJ) and complemented Δ*espJ* pICC32 (espJ pICC32) EPEC strains and then challenged with IgG-RBC for 30 min. Extracellular RBC were stained in green using Alexa™488-conjugated anti-rabbit antibodies and, after permeabilization, all cell-associated RBC were stained in red using rhodamine-conjugated anti-rabbit antibodies; cell nuclei were stained with DAPI. All bacteria are transformed with a GFP expressing plasmid. A. Representative merged images of control cells or of macrophages infected with wild-type, Δ*escN*, Δ*espF*, Δ*espJ*, and complemented Δ*espJ* pICC32 with bacteria in green (GFP), cell nuclei in blue, internalized RBC in red and extracellular RBC in yellow (merge of green and red channels). B. Quantification of phagocytosis (defined as percentage bound RBC that are internalized) and attachment index (defined as the number of RBC bound to 100 macrophages). Results are the mean ± SD of three independent experiments. Asterisks (*) denote a statistically significant difference with the wild-type strain.

In order to identify the EPEC T3SS effector responsible for the *trans*-inhibition of opsono-phagocytosis, we tested a collection of T3SS effector mutants (listed in Table 1) for their ability to inhibit FcγR-dependent phagocytosis. The striking difference in RBC phagocytosis distinguishing T3SS-competent (< 4% of the bound RBC are internalized) and T3SS-deficient (> 40% of the bound RBC are internalized) strains allowed us to make a rapid visual screen for mutants impaired in their ability to block FcγR-mediated uptake (Fig. 2A and B). Phagocytosis protects internalized RBC from antibody labelling in non-permeabilized cells, which makes them appear red under the microscope (Fig. 2A). None of the 17 effector mutant strains had any effect on the ability of IgG-opsonized RBC to bind macrophages (Fig. 2B, bottom and data not shown). The only mutant showing a significantly greater number of phagocytosed RBC than the parental strain was EPECΔ*espJ* (strain ICC190) (Dahan *et al*., 2005). Quantitative analysis revealed that infection of J774.A1 macrophages with EPECΔ*espJ* resulted in a level of RBC phagocytosis equivalent to that seen in cells infected with EPECΔ*escN* or in uninfected controls (Fig. 2B). To verify the dependency of this inhibitory activity upon EspJ, we complemented the *espJ* mutant strain ICC190 with a plasmid encoding full-length EspJ (pICC32). Expression of recombinant EspJ restored the ability of the Δ*espJ* mutant to inhibit FcγR-dependent phagocytosis to a similar level as wild-type EPEC (*P* > 0.05). These results demonstrate that EspJ is the main T3SS effector involved in the *trans*-inhibition of FcγR-mediated phagocytosis by EPEC. Interestingly, although EspF has recently been implicated in the inhibition of EPEC phagocytosis by macrophages (Quitard *et al*., 2006), we detected no statistically significant difference in the uptake of IgG-opsonized RBC by J774.A1 macrophages infected with either wild-type EPEC or the isogenic EPECΔ*espF* mutant (Fig. 2B).

**Table 1:** Bacterial strains and plasmids used in this study.

Strains/plasmids	Description	Reference
Strains		
85-170	EHEC O157:H7 spontaneous *stx*1−*stx2*−, Nal^R^	Stevens *et al*. (2004)
ICC217	Δ*escN::Kn* in 85-170, Kn^R^	This study
ICC188	Δ*espJ::Kn* in 85-170, Kn^R^	Dahan *et al*. (2005)
EDL933	EHEC O157:H7 *stx*−	ATCC
ICC187	Δ*escN::Kn* in EHEC O157:H7 strain EDL933	Garmendia *et al*. (2004)
ICC184	Δ*espF::Kn* in EHEC O157:H7 strain EDL933	Garmendia *et al*. (2004)
E2348/69	EPEC O127:H6	Levine *et al*. (1978)
ICC192	Δ*escN::Kn* in E2348/69, Kn^R^	Garmendia *et al*. (2004)
ICC211	Δ*espF::Kn* in E2348/69, Kn^R^	Marchès *et al.* (2006)
ICC190	Δ*espJ::Kn* in E2348/69, Kn^R^	Dahan *et al*. (2005)
ICC193	Δ*nleC::Kn* in E2348/69, Kn^R^	Marchès *et al*. (2005)
ICC194	Δ*nleD::Kn* in E2348/69, Kn^R^	Marchès *et al*. (2005)
ICC225	Δ*tir::Kn* in E2348/69, Kn^R^	This study
ICC257	Δ*eae::Kn* in E2348/69, Kn^R^	This study
ICC243	Δ*espG1::Kn*Δ*espG2::Cm* in E2348/69, Kn^R^ Cm^R^	This study
ICC202	Δ*map::Kn* in E2348/69, Kn^R^	Simpson *et al*. (2006)
ICC246	Δ*espH::Kn* in E2348/69, Kn^R^	This study
MK41	Δ*sepZ::AphT3* in E2348/69, Kn^R^	Kanack *et al*. (2005)
ICC249	Δ*map::Kn*Δ*espF::Cm* in E2348/69, Kn^R^ Cm^R^	This study
ICC254	Δ*nleH1::Kn*Δ*nleH2::Cm* in E2348/69, Kn^R^ Cm^R^	This study
ICC248	Δ*espI::Kn* in E2348/69, Kn^R^	This study
ICC256	Δ*nleI::Kn* in E2348/69, Kn^R^	This study
ICC252	Δ*nleF::Kn* in E2348/69, Kn^R^	This study
ICC250	Δ*nleB1::Cm* in E2348/69, Cm^R^	This study
ICC251	Δ*nleB2::Kn* in E2348/69, Kn^R^	This study
Plasmid		
pICC32	Derivative of pSA10 (Schlosser-Silverman *et al*., 2000) encoding EspJ^EPEC^–FLAG fusion protein	This study
pICC31	Derivative of pSA10 encoding EspJ^EHEC^–FLAG fusion protein	This study
pRK5-EspJ^EHEC^–FLAG	Derivative of pRK5 (BD Pharmingen) encoding EspJ^EHEC^–FLAG fusion protein	This study
pFPV25.1	Plasmid expressing *gfpmut3a* gene	Valdivia and Falkow (1996)
pSB315	Source of *aphT* cassette	Dahan *et al*. (2005)
pKD3	oriRγ, *blaM*, Cm^R^ cassette flanked by FRT sites	Datsenko and Wanner (2000)
pKD4	oriRγ, *blaM*, Kan^R^ cassette flanked by FRT sites	Datsenko and Wanner (2000)
pKD46	ori101, *repA* 101 (ts), *araBp-gam-bet-exo*, *blaM*	Datsenko and Wanner (2000)

### EspJ impairs CR3-mediated uptake

**Fig. 3:**
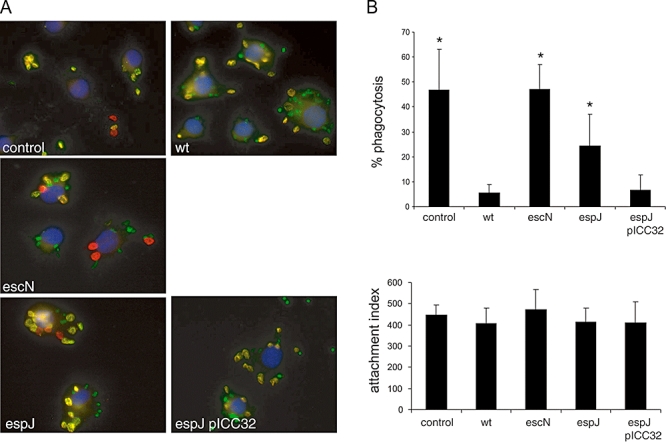
EPEC inhibition of CR3-mediated phagocytosis is EspJ dependent. J774.A1 macrophages were left uninfected (control) or infected for 1 h with primed wild-type (wt), Δ*escN* (escN), Δ*espJ* (espJ) and complemented Δ*espJ* pICC32 (espJ pICC32) EPEC strains, treated with 150 ng ml^−1^ PMA to activate the C3bi binding site on CR3 receptors, then challenged with C3bi-opsonized RBC for 30 min. Extracellular RBC were stained in green using Alexa™488-conjugated anti-rabbit antibodies and, after permeabilization, all cell-associated RBC were stained in red using rhodamine-conjugated anti-rabbit antibodies; cell nuclei were stained with DAPI. All bacteria are transformed with a GFP-expressing plasmid. A. Representative merged images of control cells and of macrophages infected with wild-type, Δ*escN*, Δ*espJ*, and complemented Δ*espJ* pICC32 with bacteria in green (GFP), cell nuclei in blue, internalized RBC in red and extracellular RBC in yellow (merge of green and red channels). B. Quantification of phagocytosis (defined as percentage bound RBC that are internalized) and attachment index (defined as the number of RBC bound to 100 macrophages). Results are the mean ± SD of three independent experiments. Asterisks (*) denote a statistically significant difference with the wild-type strain.

To study whether the antiphagocytic function of EPEC EspJ is general or specific to the FcγR-dependent signalling pathway, we examined the impact of EspJ on CR3-mediated phagocytosis. Although phagocytosis mediated by FcγR or CR3 is opsonin-dependent and actin-driven, the signalling pathways responsible for actin polymerization downstream of these two receptors are different (Caron and Hall, 1998). J774.A1 macrophages were left uninfected or infected with wild-type EPEC, EPECΔ*escN*, EPECΔ*espJ* and EPECΔ*espJ* (pICC32) (complemented), before challenge with C3bi-opsonized RBC. Wild-type EPEC inhibited phagocytosis of C3bi-opsonized RBC in a T3SS-dependent manner as no inhibition was seen after infection with EPECΔ*escN* (Fig. 3B). Deletion of *espJ* also impaired the ability of EPEC to block CR3-dependent uptake, reducing it to a level similar to EPECΔ*escN* (Fig. 3A and B). Complementing the EPECΔ*espJ* mutant restored the inhibition of C3bi-opsonized RBC uptake by infected macrophages close to wild-type EPEC levels (Fig. 3A and B). As seen for IgG-opsonized RBC, pre-infection with EPEC strains had no impact on the attachment of C3bi-opsonized RBC, suggesting that translocated EspJ does not interfere with surface expression of the phagocytic receptors (FcγR and CR3 respectively) but instead with a regulatory mechanism essential for both CR3- and FcγR-dependent uptake. These results show that EspJ inhibits opsono-phagocytosis via both the CR3 and FcγR receptors.

### EspJ from EHEC O157:H7 inhibits opsono-phagocytosis

**Fig. 4:**
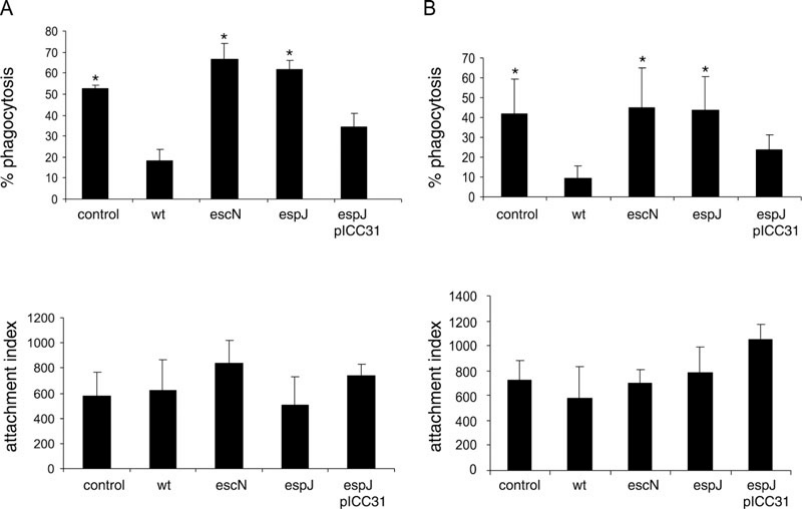
EHEC inhibits opsono-phagocytosis through the translocated effector EspJ. J774.A1 macrophages were left uninfected (control) or infected with EHEC wild-type (wt), Δ*escN* (escN), Δ*espJ* (espJ), or complemented Δ*espJ* pICC31 (espJ pICC31) for 4 h and then challenged with IgG-opsonized RBC (A) or C3bi-opsonized RBC (B) for 30 min and processed for immunofluorescence as described in *Experimental procedures*. Quantification of phagocytosis (defined as percentage bound RBC that are internalized) and attachment index (defined as the number of RBC bound to 100 macrophages) for FcγR- (A) and CR3- (B) mediated phagocytosis are shown. Results are the mean ± SD of three independent experiments. Asterisks (*) denote a statistically significant difference with the wild-type strain.

Whether or not EHEC O157:H7 have antiphagocytic activity is unknown. As *espJ* is conserved between EPEC and EHEC (Dahan *et al*., 2005), we examined whether EspJ mediates antiphagocytosis during EHEC infection of J774.A1 macrophages. J774.A1 were infected with the spontaneous *stx* minus EHEC O157:H7 strain 85-170 and its isogenic mutants EHECΔ*escN* and EHECΔ*espJ* (Table 1) before challenge with IgG- or C3bi-opsonized RBC. As shown in Fig. 4A and B, both FcγR- and CR3-dependent uptake of RBC were greatly diminished in J774.A1 infected with the parental EHEC strain compared with the levels of uptake observed in uninfected macrophages (control) or in cells infected with the EHECΔ*escN* or EHECΔ*espJ*. Complementing the Δ*espJ* strain with a plasmid encoding either EspJ^EPEC^ or EspJ^EHEC^ restored the ability of the mutant strain to inhibit phagocytosis of IgG- and C3bi-opsonized RBC (Fig. 4A and B). As for EPEC (Fig. 3), impaired RBC phagocytosis was unrelated to changes in RBC adhesion, which indicates that antiphagocytosis is due to impaired phagocytic signalling.

### EspF, not EspJ, is required for inhibition of bacterial phagocytosis

**Fig. 5:**
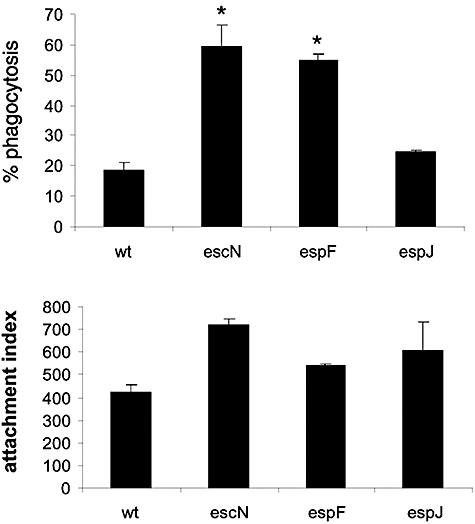
EspF, but not EspJ, controls *cis*-inhibition of EHEC phagocytosis in macrophages. J774.A1 macrophages were infected for 4 h at 37°C with 1:100 dilutions of overnight cultures of GFP-expressing EHEC O157:H7 strains as indicated and processed for immunofluorescence. Extracellular bacteria were stained red, as described in *Experimental procedures*, and were therefore easily distinguishable from internalized bacteria, which are solely green. The percentage of bound EHEC internalized (% phagocytosis) and the total number of cell-associated bacteria (attachment index) were scored under the epifluorescence microscope. Results are expressed as mean ± SD from three independent experiments, with ≥ 100 macrophages scored per condition per experiment. Asterisks (*) denote a statistically significant difference with the wild-type strain.

Several reports have involved EspF in bacterial-induced inhibition of EPEC phagocytosis by M cells and cultured macrophages (Quitard *et al*., 2006; Martinez-Argudo *et al*., 2007), a finding we have confirmed in this study (data not shown). However, no data exist on the potential inhibition of bacterial uptake by EHEC. To address this question, J774.A1 macrophages were infected for 4 h with wild-type, Δ*escN*, Δ*espJ* and Δ*espF* EHEC strains and the attachment indices and percentage phagocytosis were scored. As shown in Fig. 5, EHEC bacteria are able to interfere with their own uptake in a T3SS-dependent manner, as Δ*escN* bacteria were more readily phagocytosed than the wild-type control. Importantly, Δ*espF* EHEC behaved as the T3SS-deficient Δ*escN* isogenic control, showing that EspF^EHEC^, like EspF^EPEC^, controls *cis*-inhibition of phagocytosis. In contrast, Δ*espJ* EHEC were unimpaired in their ability to inhibit bacterial phagocytosis, establishing that two distinct molecular mechanisms control *cis*- and *trans*-inhibition of phagocytosis by EPEC and EHEC.

### EspJ expression is sufficient for antiphagocytosis in transfected Cos-7 cells

**Fig. 6:**
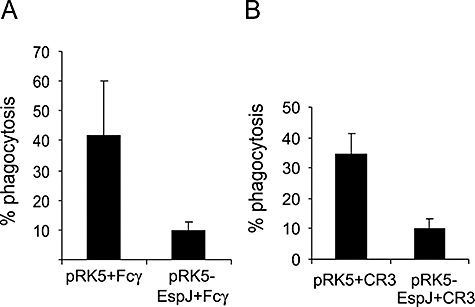
Intracellular expression of EspJ is sufficient for inhibition of FcγR- and CR3-mediated phagocytosis. Cos-7 cells were co-transfected by nucleofection with FcγRIIA (A) or CR3 receptor (B) and either with plasmid pRK5 or with pRK5-EspJ overexpressing EspJ from EHEC and were then challenged for 30 min with IgG- (A) or C3bi- (B) opsonized RBC. RBC phagocytosis was then quantified as described in *Experimental procedures*, the transfected cells being easily distinguishable from non-transfected cells by their unique ability to bind opsonized RBC. Results are the mean ± SD of at least two independent experiments.

Given the striking phenotype exhibited by *espJ* mutant strains on the inhibition of FcγR- and CR3-dependent phagocytic pathways, we examined if ectopic expression of EspJ in phagocytes was sufficient to impair opsono-phagocytosis. *espJ*^*EHEC*^ was cloned into the pRK5-FLAG eukaryotic expression vector and co-transfected with the FcγRIIA or CR3 phagocytic receptors. Cells were then challenged with IgG- or C3bi-opsonized RBC and scored for RBC binding and phagocytosis. EspJ expression had no effect on the binding of C3bi- or IgG-opsonized RBC to receptor transfected Cos-7 cells (data not shown). In contrast, a clear and statistically significant inhibition of both FcγR- and CR3-mediated uptake was observed in EspJ-expressing Cos-7 cells (Fig. 6A and B), showing that the expression of EspJ inside host cells is sufficient for inhibition of opsono-phagocytosis.

**Fig. 7:**
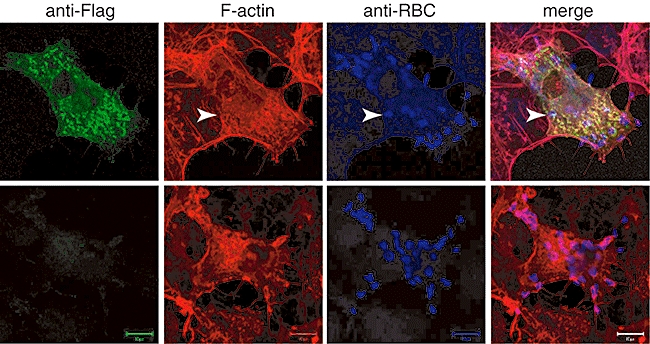
Intracellular distribution of ectopically expressed EspJ during FcγR-dependent uptake. Cos-7 cells were co-transfected with FcγRIIa and either with pRK5-EspJFLAG (top) or with empty pRK5 (bottom) and challenged with IgG-opsonized RBC for 30 min at 37°C, as described in the legend to Fig. 5. RBC-challenged cells were permeabilized and stained with an anti-flag mouse monoclonal followed by Cy2-conjugated anti-mouse antibodies (green), rhodamine phalloidin to visualize F-actin (red) and Cy5-conjugated anti-rabbit IgG-RBC (blue). Cells were observed by confocal microscopy; representative examples are shown. Scale bar, 10 μm.

The presence of a FLAG tag on EspJ allowed us to examine the basic features of the inhibition *in trans* of opsono-phagocytosis. Overexpressed EspJ was excluded from the nucleus and localized throughout the cytosol of Cos-7 cells, both in a diffuse fashion and in large aggregates. RBC challenge of Cos-7 cells coexpressing FcγRIIA and EspJ did not affect the overall localization of EspJ (Fig. 7 and data not shown). Interestingly, ectopically expressed EspJ did not specifically accumulate to any significant extent at the plasma membrane or at sites of RBC attachment. Moreover, EspJ expression did not prevent actin polymerization underneath bound IgG-opsonized RBC (Fig. 7, top, arrowhead). Overall, these data suggest that EspJ blocks FcγR-mediated phagocytosis from a distance, rather than acting locally at the nascent phagocytic cup, possibly by interfering with the later stages of RBC uptake, after initial F-actin polymerization has taken place.

## Discussion

In the past few years antiphagocytosis has been proposed as a pathogenic mechanism for EPEC (Celli *et al*., 2001). However the molecular basis of this phenomenon is poorly understood. Antiphagocytic activity is clearly dependent on the translocation of one or more effectors through the EPEC T3SS into the cytosol of macrophage-like cells and manifests itself both as an ability of EPEC to reduce their own uptake (*cis*-inhibition) and to block *in trans* the phagocytosis of IgG-opsonized zymosan particles through FcγR (*trans*-inhibition) (Goosney *et al*., 1999; Celli *et al*., 2001). Whether these two phenotypes correspond to a unique mechanism or reflects the existence of two independent antiphagocytic mechanisms was unknown. Other bacterial pathogens are known to modulate phagocytosis, either – like *Salmonella typhimurium* and *Shigella flexneri*– by stimulating their uptake by non-professional phagocytes or – like *Yersinia* and *Clostridium* spp. – by blocking phagocytic signalling in phagocytic cells. In all cases the underlying mechanisms involve the subversion of actin dynamics (reviewed in Rottner *et al*., 2005).

The mechanism by which EPEC block their own uptake has been attributed to the effector EspF (Quitard *et al*., 2006). We report in this article that EHEC O157:H7 also block their own uptake by macrophages in an EspF-dependent manner (Fig. 5), suggesting that antiphagocytosis is a general mechanism displayed by both EHEC and EPEC. In this report we demonstrate that (i) EHEC, like EPEC, are able to block the uptake of opsonized particles, (ii) EPEC and EHEC *trans*-inhibition of phagocytosis is T3SS-dependent, and (iii) EspJ (EPEC and EHEC) is the effector protein responsible for the inhibition of both FcγR- and CR3-mediated phagocytosis, suggesting that EspJ targets an essential host molecule or complex normally involved downstream of these two phagocytic receptors. Two clostridium toxins, the B toxins from *Clostridium difficile* strains 10463 and 1470, inhibit FcγR- and CR3-mediated phagocytosis (Caron and Hall, 1998; Caron *et al*., 2000). Both glucosylate and inactivate members of the Rho family of small GTP-binding proteins, known to regulate actin polymerization during a variety of eukaryotic processes, including phagocytosis (Just *et al*., 1995; Chaves-Olarte *et al*., 1999; Jaffe and Hall, 2005; Niedergang and Chavrier, 2005). However, to our knowledge, EspJ is the first example of a type three secretion effector that blocks both FcγR- and CR3-mediated phagocytosis.

Our results show that, as has been shown for EPEC, EHEC can inhibit opsono-phagocytosis. This may not be surprising, as EHEC are thought to have evolved from EPEC through the acquisition of phages encoding a Shiga-like toxin (Reid *et al*., 2000). It will be interesting to check whether other strains able to induce A/E lesions can also block phagocytosis. In order to find the effector protein conferring EPEC the ability to block phagocytosis *in trans*, we screened 17 candidate effectors mutants we had accumulated in the lab. Interestingly, neither EspF, involved in inhibition of EPEC phagocytosis by macrophages and M cells (Quitard *et al*., 2006; Martinez-Argudo *et al*., 2007); Tir, involved in redistribution of intermediate filament proteins and triggering of actin polymerization; Map, involved in filopodia formation; or EspG/EspG2, involved in disruption of the microtubule network (reviewed in Garmendia *et al*., 2005 and Caron *et al*., 2006) were involved. In contrast, deletion of *espJ*, which is carried upstream of *tccP* (Garmendia *et al*., 2004) on prophage CP-933U/Sp14 in EHEC, abolished phagocytosis of opsonized RBC. The phagocytosis defect was complemented by recombinant *espJ*. Interestingly EspJ is dispensable for *cis*-inhibition of EHEC or EPEC uptake (Fig. 5 and V. Covarelli, O. Marchès, G. Frankel and E. Caron, unpubl. results). Taken together our results show that inhibition of *cis*- and *trans*-phagocytosis are mediated by different effectors and are likely to involve different signalling pathways.

Sequence analysis reveals, as expected, that EspJ^EPEC^ shows a very strong (79%) sequence identity at the amino acid level with EspJ^EHEC^ and an open reading frame (75% identity) in *Citrobacter rodentium*, the mouse A/E pathogen (Dahan *et al*., 2005). Interestingly, database searches also show a 57% identity (74% similarity) with a putative protein from *Salmonella bongori*. *S. bongori* mainly infects cold-blooded animals but is also associated to rare cases of acute enteritis in humans (Giammanco *et al*., 2002). As *S. bongori* differs from *Salmonella enterica* by the absence of the SPI-2 (*Salmonella* pathogenicity island two), whose expression is induced intracellularly and which is essential for intracellular survival and replication within macrophages (Ochman and Groisman, 1996; Waterman and Holden, 2003), it is tempting to speculate that an antiphagocytic protein would allow *S. bongori* to survive its interaction with phagocytic cells in its hosts.

Our study establishes that EspJ is necessary and sufficient to block uptake of C3bi- and IgG-opsonized RBC. In a previous study EPEC were shown to be unable to block uptake of C3bi-opsonized zymosan (Celli *et al*., 2001). The reason for this discrepancy is unclear. While zymosan and RBC are distinct phagocytic targets, opsonization with IgG or C3bi fragments should ensure that when opsonized the two types of particles interact with identical surface receptors FcγR and CR3 respectively. The only other difference between the two studies is the source of phagocytic cells [bone marrow macrophage-derived cell line in Celli *et al*. (2001); J774.A1 and transfected Cos-7 cells in this study]. Because we obtained similar results in a murine macrophage cell line and in Cos-7 cells transfected with either FcγRIIA or CR3, and because EspJ expression is sufficient to block phagocytosis, we strongly believe that EspJ targets a critical regulatory pathway activated downstream of the two receptors; this pathway is furthermore conserved both in macrophages and in Cos-7 cells.

What is the mechanism by which EspJ blocks FcγR- and CR3-mediated opsono-phagocytosis of RBC? On the one hand, bioinformatic searches on EspJ did not yield any recognizable domain or sequence that could help us clarify its function. On the other hand, phagocytosis through these two receptors is ultrastructurally, pharmacologically and functionally different. In macrophages, FcγR-mediated uptake is constitutive and pro-inflammatory, involves the protrusion of actin-rich pseudopods and is controlled by tyrosine kinases, Cdc42 and Rac activity; in contrast, CR3-dependent internalization is not accompanied by major protrusions or production of pro-inflammatory signals, does not require tyrosine kinase, Rac or Cdc42 activity but is dependent on RhoA activity for actin polymerization at sites of particle binding (Allen and Aderem, 1996; Caron and Hall, 1998; Niedergang and Chavrier, 2005). Nonetheless, these two modes of engulfment share two requirements: local actin polymerization and membrane delivery at sites of particle binding (Allen and Aderem, 1996; Braun and Niedergang, 2006). We show that F-actin is still detected at sites of RBC binding in EspJ-expressing cells, suggesting that EspJ could interfere with the delivery of membrane at nascent phagosomes. In line with this observation, EspJ is found on intracellular structures, possibly endomembranes, when ectopically expressed in Cos-7 cells and is not recruited to forming phagosomes. Phagocytosis is known to involve the focal delivery of membrane from various intracellular sources at sites of particle binding (Gagnon *et al*., 2002; Braun *et al*., 2004). Identification of the putative EspJ-rich compartment would help us shed light on the EspJ-dependent antiphagocytic mechanism, which might involve the blocking either of membrane *per se* or of some unknown membrane-borne regulator(s) of uptake.

Current understanding of infections by A/E pathogens points towards an extracellular lifestyle, with bacteria adhering strongly to the surface of enterocytes. In EPEC and EHEC, type three secretion, which controls formation of A/E lesions, is important for host colonization (reviewed in Spears *et al*., 2006). It is now clear that the T3SS also controls antiphagocytosis (Goosney *et al*., 1999; Celli *et al*., 2001; this report). Importantly, ultrastructural studies showed that EHEC O157:H7 colonize Peyer's patch mucosa *ex vivo* (Phillips *et al*., 2000), *C. rodentium* first targets the caecal patch *in vivo* (Wiles *et al*., 2004) while RDEC-1 (rabbit EPEC) first targets ileal M cells, an intestinal phagocyte that binds but does not internalize these bacteria, before spreading to other intestinal sites (Inman and Cantey, 1983). It is conceivable that EspF-mediated, *cis*-inhibition of phagocytosis (Quitard *et al*., 2006; Martinez-Argudo *et al*., 2007) allows A/E bacteria to prevent their internalization early on during infection and thereby facilitates colonization of the intestine. EspJ-mediated *trans*-inhibition could be a mechanism to ensure that bacteria-associated host cells are not internalized by phagocytic cells that are recruited to the lumen of the gut as a result of inflammation once colonization of the epithelial is established (Inman and Cantey, 1983) or after an adaptive immune response has been mounted. Further studies are needed to unravel the molecular basis of EspJ and EspF antiphagocytic activities and their respective roles in colonization and infection.

## Experimental procedures

### Bacterial strains

The wild-type strains EPEC O127:H6 E2349/68 and EHEC O157:H7 85-170 used in this study and their mutants are listed in Table 1. Bacteria were grown in Luria–Bertani (LB) medium or in DMEM supplemented with kanamycin (50 μg ml^−1^), chloramphenicol (5 μg ml^−1^) and carbenicillin (100 μg ml^−1^), when necessary. The mutant strains engineered during this study were constructed using the PCR one-step λ Red recombinase method (Datsenko and Wanner, 2000). Briefly, each mutation was obtained using a PCR product containing an antibiotic resistance gene flanked by the 50 bases from the 5′ and 3′ ends of the target gene. Plasmids pKD4, pKK3 and pSB315 were used as PCR template. The PCR products were electroporated into the recipient strains carrying the Red system expression plasmid pKD46 and mutants were selected on LB plates with kanamycin or chloramphenicol. Recombinant clones were cured of pKD46 plasmid by growth at the non-permissive temperature (42°C) and mutation confirmed by different PCR reactions using primers flanking the targeted region and primers into the antibiotic resistance gene.

### Cell culture and transfection

Cells from the murine macrophage J774.A1 and simian kidney fibroblast Cos-7 cell lines (ATCC) were maintained in DMEM supplemented with 10% heat-inactivated fetal calf serum (FCS) and penicillin/streptomycin. J774.A1 were seeded on glass coverslips (13 mm diameter) in 24-well plates at a density of 5 × 10^4^ cells per well 24 h before infection. Cos-7 cells were seeded on coverslips in 6 cm dishes (10^5^ cells per dish) and transfected either by nucleofection (Amaxa, Cologne, Germany) or using the calcium/phosphate protocol (Cougoule *et al*., 2006). Briefly, DNA/calcium phosphate precipitates (10 μg of DNA/400 μl of calcium phosphate/6 cm dishes containing 3.6 ml of fresh medium) were added onto the cells for 16–18 h, washed and incubated in fresh medium for an additional 6 h.

### Plasmids

pICC32 and pICC31 are derivatives of pSA10 (Schlosser-Silverman *et al*., 2000), a vector containing multiple cloning sites downstream of the *tac* promoter. Pair of primers EspJf1 5′-CGGAATTCATGCCAATCATAAAGAACTGC-3′ and EspJr1 5′-AAAACTGCAGTTATTTATCATCATCATCTTTATAATCTTTTTTGAGTGGGTGGATAT-3′ and pair of primers EspJf2 5′-CGGAATTCATGTCAATTATAAAAAACTGCTTATC-3′ and EspJr2 5′-AAAACTGCAGTTATTTATCATCATCATCTTTATAATCTTTTTTGAGAGGATATATGTCAAC-3′ were used to amplify *espJ* fused to a FLAG tag from the wild-type EPEC and the wild-type EHEC respectively. PCR products containing terminal EcoRI and PstI restriction sites were digested and cloned into pSA10, generating plasmids pICC32 and pICC31. Plasmid pRK5-EspJ^EHEC^FLAG encoding EspJ from EHEC fused to a FLAG tag was obtained by EcoRI and PstI digestion of the PCR fragment obtained with primers EspJf2–EspJr2 and cloning into the eukaryotic expression vector pRK5 (BD Pharmingen).

### Phagocytic assay and immunofluorescence

Overnight EPEC cultures in LB were diluted 1:100 into DMEM containing 25 mM Hepes and 2 mM Glutamax (Invitrogen) and pre-activated by incubation for 3 h at 37°C in a 5% CO_2_ atmosphere before infection. EHEC bacteria were grown overnight in DMEM supplemented with 5% FCS and directly added onto mammalian cells. J774.A1 or Cos-7 cells were incubated for at least 1 h in serum-free medium then infected either for 1 h with EPEC at a multiplicity of infection (moi) of 20:1 or for 4 h with EHEC at a moi of 200:1, conditions that lead a similar average number of bacteria (20–50) interacting with each macrophage at the end of the infection period. Conditions for optimal induction of LEE expression and optimal association with host cells have been described previously (Garmendia *et al*., 2004; Quitard *et al*., 2006).

#### RBC phagocytosis

Monolayers of infected cells were then washed with PBS and challenged for 30 min at 37°C in 5% CO_2_ with 500 μl of IgG- or C3bi-opsonized sheep RBC (TCS) at a ratio of 30 RBC per cell. RBC were opsonized as previously described (Caron and Hall, 1998; Patel *et al*., 2002). Briefly, for FcγR-mediated phagocytosis, 0.5 μl of RBC per coverslip were opsonized for 30 min with a subagglutinating concentration of rabbit anti-RBC IgG (Cappel) in 1 ml of gelatin veronal buffer (GVB^2+^, Sigma), washed once with GVB^2+^ and re-suspended in 500 μl of DMEM/coverslip. For CR3-mediated phagocytosis, 0.5 μl of RBC per coverslip were opsonized with rabbit anti-RBC IgM (Cedarlane Laboratories) for 30 min at room temperature in 1 ml of GVB^2+^, pelleted, incubated for 20 min at 37°C with C5-deficient human serum (Sigma) and re-suspended in 500 μl of DMEM/coverslip. For efficient binding and phagocytosis of C3bi-opsonized RBC, J774.A1 were pre-treated with 150 ng ml^−1^ PMA in serum-free DMEM for 15 min before RBC challenge. After phagocytic challenge, cells were washed twice in PBS and fixed for 20 min in 4% paraformaldehyde. Free aldehyde groups were neutralized with 50 mM NH_4_Cl in PBS for 15 min and cells were blocked with 10% donkey serum in PBS for 30 min. For differential staining of external and internalized IgG- or C3bi-opsonized RBC, cells were incubated with Alexa™488-conjugated donkey anti-rabbit antibodies, permeabilized with 0.5% Triton for 10 min and incubated with Rhodamine-conjugated donkey anti-rabbit antibodies. Coverslips were mounted in Pro-Long antifade reagent (Invitrogen) and analysed using a ZEISS Axioimager fluorescence microscope. Binding and phagocytosis indices were determined by counting the number of RBC, respectively, associated to and internalized by a minimum of 50 cells, be they J774A.1 macrophages, FcγR- or CR3-transfected Cos-7 cells.

#### EHEC phagocytosis

EHEC bacterial strains expressing the GFP-encoding pFPV25.1 plasmid were used (see Table 1). Overnight cultures were diluted 1/100 in SFM supplemented with 25 mM Hepes and the appropriate antibiotic, then added onto J774.A1 macrophages for 4 h at 37°C. Cells were fixed and incubated successively with goat anti-EHEC O157:H7 IgG (Fitzgerald) then Texas red-conjugated donkey anti-goat IgG, to stain extracellular EHEC in red. Cells were then permeabilized, F-actin was labelled red, using Rhodamine RedX-coupled phalloidin (Molecular Probes), and coverslips were mounted on Mowiol (Calbiochem). The numbers of bacteria bound (red) and internalized (green) were scored using an epifluorescence microscope (BX50, Olympus). The levels of phagocytosis measured for wild-type and Δ*escN* EHEC were equivalent for the 85-170 and EDL-933 EHEC strains.

All data were analysed by unpaired, two-tailed Student's *t*-test, and considered statistically significant for *P* < 0.05.
